# Preliminary experience on the safety and tolerability of mechanical “insufflation-exsufflation” in subjects with artificial airway

**DOI:** 10.1186/s40635-018-0173-6

**Published:** 2018-04-03

**Authors:** Miguel Sánchez-García, Passio Santos, Gema Rodríguez-Trigo, Fernando Martínez-Sagasti, Tomás Fariña-González, Ángela del Pino-Ramírez, Carlos Cardenal-Sánchez, Beatriz Busto-González, Mónica Requesens-Solera, Mercedes Nieto-Cabrera, Francisco Romero-Romero, Antonio Núñez-Reiz

**Affiliations:** 1 Critical Care Department, Hospital Clínico San Carlos, c/Prof. Martín Lagos s/n, 28040 Madrid, Spain; 2 Physiotherapy and Rehabilitation Service, Hospital Clínico San Carlos, c/Prof. Martín Lagos s/n, 28040 Madrid, Spain; 3 Service of Pulmonology, Hospital Clínico San Carlos, c/Prof. Martín Lagos s/n, 28040 Madrid, Spain

**Keywords:** Mechanical insufflation-exsufflation, Airway clearance, Safety, Artificial airway, Endotracheal intubation, Respiratory tract secretions, Secretion suctioning, Endotracheal aspiration

## Abstract

**Background:**

Catheter suctioning of respiratory secretions in intubated subjects is limited to the proximal airway and associated with traumatic lesions to the mucosa and poor tolerance. “Mechanical insufflation-exsufflation” exerts positive pressure, followed by an abrupt drop to negative pressure. Potential advantages of this technique are aspiration of distal airway secretions, avoiding trauma, and improving tolerance.

**Methods:**

We applied insufflation of 50 cmH_2_O for 3 s and exsufflation of − 45 cmH_2_O for 4 s in patients with an endotracheal tube or tracheostomy cannula requiring secretion suctioning. Cycles of 10 to 12 insufflations-exsufflations were performed and repeated if secretions were aspirated and visible in the proximal artificial airway. Clinical and laboratory parameters were collected before and 5 and 60 min after the procedure. Subjects were followed during their ICU stay until discharge or death.

**Results:**

Mechanical insufflation-exsufflation was applied 26 times to 7 male and 6 female subjects requiring suctioning. Mean age was 62.6 ± 20 years and mean Apache II score 23.3 ± 7.4 points. At each session, a median of 2 (IQR 1; 2) cycles on median day of intubation 11.5 (IQR 6.25; 25.75) were performed. Mean insufflation tidal volume was 1043.6 ± 649.9 ml. No statistically significant differences were identified between baseline and post-procedure time points. Barotrauma, desaturation, atelectasis, hemoptysis, or other airway complication and hemodynamic complications were not detected. All, except one, of the mechanical insufflation-exsufflation sessions were productive, showing secretions in the proximal artificial airway, and were well tolerated.

**Conclusions:**

Our preliminary data suggest that mechanical insufflation-exsufflation may be safe and effective in patients with artificial airway. Safety and efficacy need to be confirmed in larger studies with different patient populations.

**Trial registration:**

EudraCT 2017-005201-13 (EU Clinical Trials Register).

## Background

Aspiration of respiratory secretions is a frequently needed procedure in intubated subjects, which is performed whenever respiratory secretions accumulate. The conventional method consists of inserting a sterile catheter through the endotracheal tube or tracheostomy cannula connected to a negative pressure port into the trachea until resistance is met [1]. Its effect is therefore limited to the trachea and carina and efficacy is reduced in the presence of dense secretions. Frequent complications include traumatic lesions to the mucosa, poor tolerance [2], and pain [3], as well as, mostly transient, respiratory and hemodynamic adverse events [4, 5]. Airway clearance devices providing mechanically assisted cough or mechanical insufflation-exsufflation (MIE), in contrast to manually assisted cough, are being used to facilitate aspiration of tracheobronchial secretions. They simulate the physiological cough mechanism by applying positive pressure to the airway followed by an abrupt decrease to a negative pressure [6]. Experience with non-invasive secretion clearance with MIE has accumulated in non-critically ill patients with neuromuscular diseases (NMD), both chronic ventilator-dependent [7–9] and spontaneously breathing subjects [8, 10], hospitalized or at home [11]. The specific indication for the use of a mechanical airway clearance device is the lack of effective cough in spite of respiratory physiotherapy, with the aim to reduce the risk for atelectasis and respiratory tract infection. The usual interface is a face mask, although the intended use of the devices also includes patients with endotracheal intubation [6, 12].

Compared to conventional secretions suctioning with a sterile catheter inserted through the endotracheal tube or tracheostomy cannula, potential advantages of MIE are a more effective aspiration of distal airway secretions, avoidance of direct trauma to the airway mucosa, and improved tolerance of the aspiration maneuver. On the other hand, however, the short “recruitment” maneuver during the positive pressure phase may increase the risk of respiratory and hemodynamic complications [6, 13–15]. The few published experiences reporting on its use in intensive care do not report complications associated with the use of MIE [10, 16–18], although concerns about the safety of the technique in critically ill patients have been voiced [19, 20]. The technique has been introduced in clinical practice in our department to support respiratory tract secretion suctioning in subjects needing frequent suctioning, to facilitate weaning and/or to reduce the risk of reintubation. We report on our initial experience in evaluating the safety of MIE in an unselected intubated patient population of a broad range of severity.

## Methods

We report on our experience in a short series of patients in whom MIE was first used. Cases with endotracheal or tracheostomy tubes requiring aspiration of tracheobronchial secretions were selected, irrespective of degree of respiratory failure, hemodynamic instability or other organ dysfunctions, primary or secondary acute lung dysfunction, atelectasis, tracheobronchitis, pneumonia, and intracranial hypertension. The only inclusion criterion for aspiration of respiratory secretions with the “Cough Assist™” airway clearance device was therefore the need for secretion suctioning, regardless of severity or degree of respiratory or other organ dysfunctions.

Subjects on any assisted mode of ventilation requiring aspiration of secretions by clinical judgment or ventilator display curves [21] were connected to a mechanical aspiration device (Cough Assist E70™, Respironics, Philips) without previously performing conventional secretion suctioning. Insufflation plateau was set at 50 cmH_2_O, with a duration of 3 s, followed by an exsufflation phase at a negative pressure of − 45 cmH_2_O maintained for 4 s. These insufflation-exsufflation pressures are slightly above the ± 40 cmH_2_O recommended by the manufacturer, which are meant to be applied by face mask mainly, and were chosen to overcome resistance to air flow exerted by the artificial airways [22]. The device also applies oscillation at 16 Hz during both insufflation and exsufflation plateaus. The synchronized, i.e., patient-triggered “cough track” mode with “high” inhale flow was programmed for all 26 sessions. Study subjects were not instructed to exhale or try to cough during the exsufflation phase.

**Fig. 1:**
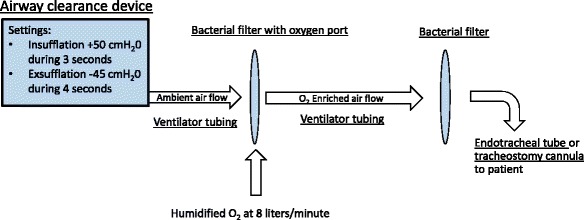
Schematic of the setup of the mechanical insufflation-exsufflation procedure

Cycles of 10 to 12 MIE were applied and only repeated if productive. A cycle was defined as productive if secretions were visible in the proximal segment of the artificial airway at the 10th to 12th insufflation-exsufflation. Secretions aspirated to the lumen of the proximal segment of the endotracheal tube or tracheostomy cannula were aspirated with a sterile catheter with the tip remaining in the proximal artificial airway. A tubing with bacterial heat-moisture exchange filters (Intersurgical “Inter-Therm HMEF” with luer port) at both ends was used to attach the device to the artificial airway, with an oxygen flow of 8 l/min connected to the luer port of the filter close to the MIE device (Fig. 1).

Clinical and biochemical variables were collected at baseline, immediately before MIE, and 5 and 60 min after the sessions. We recorded ventilator modes and parameters, arterial or venous blood gases, and hemodynamic parameters. Half of the cases had an arterial catheter inserted.

Subjects were disconnected from the ventilator tubing to be connected to the tubing of the airway clearance device. Immediately after MIE, mechanically ventilated and spontaneously breathing subjects were returned to their baseline ventilator status, without any change in settings, mode, or inspiratory fraction of oxygen (FiO_2_).

Subjects were followed for complications potentially related to MIE during their ICU stay until discharge or death, including review of daily chest X-rays.

The approved use of CoughAssist™ includes secretion suctioning in subjects with artificial airway [6, 12] and is available and commonly applied in our critical care department.

### Statistical analyses

Data were entered in a Microsoft Excel spreadsheet. Quantitative variables collected at baseline and 60 min after the procedure or at baseline and the 5- and 60-min time points were compared by Student’s *t* test, analysis of variance for repeated measurements, or non-parametric tests, as appropriate. Qualitative variables were compared using the chi-square or Fisher tests. Baseline ventilator status was only compared to 60 min post-intervention parameters, i.e., without the 5-min post-intervention data, because, per protocol, subjects were returned to baseline parameters immediately after MIE. We did not perform a sample size calculation but rather chose to study a small number of subjects exposed to our routine MIE parameters under close clinical and laboratory monitoring conditions to maximize safety.

## Results

We studied 13 subjects, 7 males and 6 females, with a mean age of 62.6 ± 20 years and a mean Apache II score at ICU admission of 23.3 ± 7.4 points, who were connected to the airway clearance device a total of 26 times on median day 11.5 (IQR 6.25; 28.25) after ICU admission and 11.5 (IQR 6.25; 25.75) after endotracheal intubation. Per session, a median of 2 (IQR 1; 2) cycles were performed. The device was connected 16 times to a tracheostomy tube and 10 times to an endotracheal tube. The characteristics of subjects are listed in Table 1.

**Table 1:** Patient characteristics and baseline parameters

Patient	Sex	Age (years)	Apache II (at ICU admission)	ICU stay at first study day	Days of intubation at first study day	Diagnostic group	Main admission diagnoses	Main comorbidity	PaO2/FiO2 (mmHg) or % Sat	PEEP (cmH_2_O)	ETT/T sizes (mm)
1	F	46	28	17	16	Postop	Peritonitis	Colon cancer	366	6	8
2	M	68	18	52	41	Medical	Severe pancreatitis	Hypertension	100%	5	8
3	M	82	14	6	5	Postop	Nosocomial pneumonia	Hypertension, CAD, CHF	218	5	8
4	M	82	21	11	11	Postop	Cardiac arrest	CABG, CHF, asthma	233	0	8
5	F	54	18	1	1	Medical	Respiratory failure	Poliomyelitis, kyphoscoliosis	276	5	7
6	F	18	23	36	36	Medical	Coma	Viral encephalitis	94%	7	7
7	M	26	19	3	3	Medical	Coma	Head trauma	128	7	8
8	F	69	25	7	7	Medical	Coma	Subarachnoid hemorrhage	128	10	8
9	M	51	17	26	26	Medical	Coma	Ischemic stroke, intracranial hypertension, craniectomy	96%	5	8
10	M	75	38	1	1	Medical	Severe CAP	Hypertension, stroke, leukemia	182	12	8.5
11	M	80	28	6	6	Trauma	Coma	Head trauma	98%	5	8
12	F	61	18	8	8	Medical	Bronchospasm	COPD, alcohol abuse, diabetes mellitus, chronic hepatitis and pancreatitis	120	7	8
13	F	80	36	11	11	Medical	Cardiac arrest	Complete atrio-ventricular bloc	270	6	7

*CAD* coronary artery disease, *CHF* congestive heart failure, *CABG* coronary artery bypass graft, *CAP* community-acquired pneumonia, *COPD* chronic obstructive pulmonary disease, *PaO2/FiO2* ratio of partial pressure of oxygen in arterial blood to fraction of inspired oxygen, *%Sat* pulse-oxymetry saturation, *PEEP* positive end-expiratory pressure, *ETT/T* endotracheal tube/tracheostomy cannula inner diameter in mm

One patient with a baseline intracranial pressure of 17 cmH_2_O showed an increase to 28 cmH_2_O during the 3 s of the first mechanical insufflation, with pressures remaining around 0 in the second and all subsequent MIE cycles and an intracranial pressure of 15 cmH2O at 60 min after the procedure. Another patient, under extracorporeal membrane oxygenation for a bronchopleural fistula developing after major heart surgery, also suffering from hospital-acquired pneumonia, was being ventilated with plateau airway pressures below 20 cmH_2_O. MIE pressures were therefore reduced to + 20 and − 40 cmH_2_O, respectively, in this patient. No pleural air leak was observed during the procedure, while abundant purulent secretions were aspirated.

Table 2 shows hemodynamic and respiratory parameters collected at baseline and 5 and 60 min after MIE, without statistically significant differences between time points for any variable, except for a significant increase of pulse oximetry oxygen saturation and PaO_2_.

**Table 2:** Baseline and follow-up respiratory and hemodynamic parameters

	Baseline	5′	60″	*p*
Temperature, °C	37.3 ± 0.82		37.4 ± 0.7	ns
Heart rate, bpm	97.7 ± 14.4	97.1 ± 15.1	97.3 ± 14.2	0.82
Mean blood pressure, mmHg	87 ± 11.9	87.6 ± 14.7	83.6 ± 14.7	0.57
Sat O_2_, %	97.38 ± 2.37	98.3 ± 2.2	97 ± 3.4	0.04
pHa	7.43 ± 0.1	7.38 ± 0.1	7.45 ± 0.05	0.17
pHv	7.39 ± 0.1	7.44 ± 0.02	7.42 ± 0.05	0.13
PaO_2_, mmHg	105.5 ± 23.9	124 ± 55.5	*143 ± 42.3	0.031
PvO_2_, mmHg	54.9 ± 37.4	34.5 ± 4.9	52.4 ± 29	0.67
PaCO_2_, mmHg	37.5 ± 12.6	40.5 ± 13.3	35.2 ± 8.2	0.058
PvCO_2_, mmHg	45.2 ± 6.7	44.5 ± 3.5	40.2 ± 5.2	0.15
Ventilator mode				0.61
CPAP PC-IMV	20		18	
MMV VC-IMV	2		1	
Spontaneous respiration	4		7	
FiO_2_	0.46 ± 0.13	0.45 ± 0.13	0.44 ± 0.1	0.73
PaO_2_/FiO_2_	239.8 ± 96.8	285.5 ± 140	328.7 ± 104.7	0.14
PvO_2_/FiO_2_	134.9 ± 114.2	105.9 ± 3.7	132.6 ± 93.9	0.96
Total breath, rate/min	22.1 ± 6.3	23.5 ± 7.1	20.7 ± 5.9	0.10
Minute volume, l/min	10.6 ± 3.6	11.0 ± 3.2	9.6 ± 2.6	0.13
Tidal volume, ml	524.1 ± 106.6	496.3 ± 118.4	493.2 ± 139	0.15
PEEP, mmHg	5.3 ± 2.9	5.3 ± 2.9	4.6 ± 3.2	0.45
Peak inspiratory pressure, cmH_2_O	23.6 ± 7.8	24.0 ± 8.1	22.3 ± 5.6	0.17
Plateau pressure, cmH_2_O	21.9 ± 5.1	22.5 ± 5.4	20.9 ± 4.4	0.14
Lung compliance				
Dynamic	17.7 ± 9.5	16.3 ± 9.4	17.6 ± 9.6	0.4
Static, ml/cmH_2_O	18.7 ± 8.8	17.2 ± 9.1	19.1 ± 9.3	0.33
CA tidal volume, ml	1043.6 ± 649.9			

*Bpm* beats per minute, *Sat O*_*2*_ pulse-oximetry oxygen saturation, *pHa* arterial pH, *pHv* venous pH, *PaO*_*2*_ arterial oxygen partial pressure, *PvO*_*2*_ venous oxygen partial pressure, *PaCO*_*2*_ arterial CO2 partial pressure, *PvCO*_*2*_ venous CO_2_ partial pressure, *CPAP* assisted pressure ventilation, *MMV* minimal mandatory ventilation, *BiPAP* controlled pressure mandatory ventilation, *IPPV* volume-targeted pressure control ventilation, *PEEP* positive end-expiratory pressure, *CA* Cough Assist

*Significant difference from baseline

MIE was considered successful, i.e., respiratory tract secretions were aspirated and visible in the proximal segments of the artificial airway, in all except one patient, in whom no secretions were evidenced. The procedure was well tolerated in all cases, and no additional sedatives or analgesic medications or changes in vasopressor infusion rates were required. No barotrauma, i.e., pneumothorax, nor atelectasis were observed on daily follow-up chest X-rays. No patient developed desaturation, suggesting de-recruitment, or hemoptysis or other airway complications.

## Discussion

The data presented here are a first step in the evaluation of the safety and efficacy of mechanical insufflation-exsufflation (MIE) in subjects with artificial airway. We did not detect adverse events occurring during or after the procedure potentially related to MIE with the parameters employed in this cohort of subjects. Recent versions of airway clearance devices allow for application of insufflation and exsufflation, i.e., positive and negative pressures, of up to 70 and − 70 cmH_2_O, respectively, with duration of inspiratory and expiratory plateau pressures of up to 5 s and optional concomitant high frequency oscillation during both. For this initial approach to the technique, we chose to apply our routine, more conservative, + 50 and − 45 cmH_2_O and 3 and 4 s duration settings. We also usually provide continuous oxygen flow during the procedure because the airway clearance device uses ambient air without supplemental oxygen.

The significant increase of partial arterial oxygen pressure observed after MIE may be due to the administration of a continuous flow of oxygen at 8 l/min at the filter port adjacent to the artificial airway and to the clearance of airway secretions with a concomitant recruitment effect of 3 s of positive pressure at 50 cmH_2_O. If the latter is true, it could have contributed to avoid de-recruitment due to disconnection from positive pressure mechanical ventilation, as is the case for the conventional secretion suctioning procedure. The effect of the short 3-s + 50-cmH_2_O “recruitment maneuver” is supported by the observed increase of arterial oxygenation from 5 to 60 min after MIE. Thus, in our short series of critically ill intubated patients, disconnection did not result in loss of positive pressure, alveolar collapse, and desaturation [19, 20]. Conversely, conventional suctioning implies disconnection and drop of airway pressure, as well as interruption of ventilation and oxygenation. In the only randomized trial comparing MIE with the conventional suctioning procedure in critically ill intubated patients [16], most of the complications actually occurred in the control group, except for one patient in the study group developing hypotension. Blood pressure may have remained stable in our cohort because of our patients having an adequate intravascular volume status, although we did not monitor any direct parameter to measure it. Additionally, as the reduction in venous return due to positive thoracic pressure during the first insufflation is immediately compensated by the negative thoracic exsufflation pressure, which increases venous return, fluctuations in blood pressure may not be clinically detected or relevant.

Gastric distension is a complication reported only in non-intubated patients with NMD, where MIE is applied by face mask. In a previous study, 2 out of 11 subjects developed stomach distension, with one being complicated by gastroesophageal reflux requiring endotracheal intubation [10].

The importance of our preliminary data on secretion suctioning relates to the scarcity of studies concerning MIE in acute care settings that have been published. The role of MIE is well established in neuromuscular disease patients in chronic and acute settings but not as a routine in  critical care. The few published experiences reporting on the use of MIE in intensive care include a single randomized study showing a significant reduction of reintubation when employing MIE immediately before and during 48 h after extubation [16]. The combination of post-extubation non-invasive mechanical ventilation with pre- and post-extubation MIE was associated with successful extubation in a population of 157 “unweanable” patients, 89% of whom suffered from NMD [18]. Another publication describes a single case of traumatic quadriplegia successfully weaned from mechanical ventilation supported in part by MIE applied immediately after extubation [17]. A small study with a historical control group showed a reduction in the number of subjects needing endotracheal intubation for invasive mechanical ventilation in a group of patients with NMD admitted to intensive care for acute respiratory insufficiency with or without respiratory tract infections, in whom MIE was applied with a face mask [10]. An analogy between patients suffering from NMD and the critical ill who develop polyneuropathy and myopathy [23], both unable to cough their respiratory tract secretion, can be established to justify further investigation of MIE in critically ill intubated patients.

An additional reason for further assessing the safety and efficacy of MIE is the clinical relevance and frequency of the main adverse events associated with conventional suctioning techniques, as well as its limited effectiveness. The tip of the catheter exerts negative pressure only within a small area of the proximal airway, due, at least in part, to lack of proximal sealing. In addition, repeated insertion of the suctioning catheter causes traumatic injury to the mucosa. Tolerance of endotracheal aspiration is poor and reported to constitute one of the most painful maneuvers in critically ill patients [3], and administration of analgesia, with or without sedatives, prior to the maneuver has been advocated [2]. Finally, a frequently observed complication is desaturation associated with disconnection from the ventilator, particularly in patients with severe respiratory insufficiency and high PEEP settings. Although our preliminary findings suggest that MIE may achieve airway clearance at a reduced morbidity cost, several limitations need to be acknowledged. The main caveats of our study results are the small sample size and the relative clinical stability of most patients, thus preventing us from drawing conclusions for patients in higher severity groups, like those with ARDS or severe hemodynamic dysfunction. Also, not being a controlled study, the observed safety and efficacy data need to be further evaluated in future randomized controlled trials.

## Conclusions

The potential benefits of MIE, beyond airway clearance during weaning and avoiding reintubation [16], remain to be proven and potentially include reduction of adverse events and improved tolerance, as well as prevention and/or treatment of respiratory tract infection in intubated patients. The current report only provides data of clinical practice and, therefore, as a first step, safety of the technique needs to be confirmed over a broader range of severity and in studies of comparative randomized design.

## Abbreviations

Apache II: Acute Physiology and Chronic Health Evaluation
FiO2: Inspiratory fraction of oxygen
ICU: Intensive care unit
IQR: Interquartile range
MIE: Mechanical insufflation-exsufflation
NMD: Neuromuscular diseases
